# Revision rate is higher in patients with periprosthetic femur fractures following revision arthroplasty in comparison with ORIF following our algorithm: a two-center 1 analysis of 129 patients

**DOI:** 10.1007/s00068-021-01832-8

**Published:** 2021-11-12

**Authors:** Patrick Pflüger, Eftychios Bolierakis, Markus Wurm, Klemens Horst, Frank Hildebrand, Peter Biberthaler

**Affiliations:** 1 Department of Trauma Surgery, Klinikum Rechts Der Isar, Technical University of Munich, Ismaninger Str. 22, 81675 Munich, Germany; 2 Department of Orthopaedics, Trauma and Reconstructive Surgery, University Hospital RWTH Aachen, Aachen, Germany

**Keywords:** Periprosthetic fracture, Femoral fracture, Hip arthroplasty, Open reduction internal fixation, Revision arthroplasty

## Abstract

**Purpose:**

Effective therapy of periprosthetic femur fractures of the hip (PPF) are challenging due to patients’ frailty and complexity of fracture patterns. The aim of this cohort study was to analyze the radiological and functional outcome following PPF.

**Methods:**

A retrospective, multicenter study in the period 2009–2019 of patients with PPF at two level I trauma centers in Germany was performed. PPF were classified according to the Vancouver classification system. Demographic data, American Society of Anesthesiologists (ASA) classification, type of surgery, complications, and reoperation rate were obtained from patient records. The functional outcome was assessed by the modified Harris-Hip Score (mHHS), general health using the EQ-5D, and radiological outcome by Beals & Tower (B&T) criteria.

**Results:**

A total of 129 patients with a mean age of 79 years (range 43–102) were included. 70% of all patients were female and 68% of the patients had an ASA score ≥ 3. 20 patients suffered from a Vancouver A, 90 from a Vancouver B and 19 from a Vancouver C fracture. 14% of the patients died within the first 2 years after surgery. The reoperation rate after open reduction and internal fixation (ORIF) (*n* = 60) was 8% and after revision arthroplasty (RA) (*n* = 47) 30% (OR 3.4, 95% CI [1.21–10.2]). Mean mHHS (*n* = 32) was 53 ± 19.4 and EQ-VAS was 50 ± 24.6. According to B&T criteria, 82% of patients treated with ORIF (*n* = 17) and 62% after RA (*n* = 13) showed an excellent outcome.

**Conclusion:**

Patients with a PPF of the hip are elderly and at increased operative risk. In cases with a stable prosthesis, ORIF provides good radiological outcome with low reoperation rates. In case of RA, the risk for revision surgery is higher.

## Background

Total hip arthroplasties (THA) represent one of the most common orthopaedic procedures with increasing numbers in the last decades [1]. As a result of this development, the number of periprosthetic fractures of the hip (PPF) is increasing and constitute the second most frequent reason for revision surgery [2, 3]. The management of PPFs is challenging due to the complex patient population and required medical expertise. Typically, patients are old and often suffer from several comorbidities. This contributes to a limited functional demand and 1-year mortality rates up to 22% [4–6]. Effective management of these injuries requires a multidisciplinary approach as well as advanced surgical skills. In this context, advanced trauma and arthroplasty skills are crucial for the surgical management since preoperative radiographs alone can be inconclusive [7, 8]. Therefore, the treatment of PPFs should be carried out in major orthopaedic trauma centers with experienced medical personal and a multidisciplinary setting.

The treatment of PPF of the hip is currently guided by the most widely utilized Vancouver classification system [9, 10]. A broad consensus exists regarding the treatment of Vancouver A and C fractures, but there is an ongoing discussion about the validity of the Vancouver B subtypes and, therefore, new classification systems were developed [10–12].

Due to the complex and challenging management of these injuries with high mortality rates and limited follow-up, literature still does not provide robust data [10, 13]. In this respect, the aim of the presented study was to analyze the outcome of patients treated for a periprosthetic femur fracture of the hip in two level I orthopaedic trauma centers.

## Patients and methods

### Patients

A retrospective, multicenter analysis from 2009 to 2019 was performed in two level I orthopaedic trauma departments (Munich and Aachen, Germany). The study was approved by local regulatory committees (No: 313/18 s, Technical University of Munich, Germany and No: EK272/19, RWTH Aachen, Germany) as a retrospective cohort study with cross-sectional data.

Inclusion criteria: patients with a femoral PPF after hip arthroplasty, age > 18 years, who were capable of giving informed consent and underwent treatment at one of the two study centers. Patients’ and THA history, as well as biochemical markers, pre- and intra-operative clinical findings indicating the presence of infection were assessed and if any suspicion they were excluded from the study group.

### Primary endpoints

Primary study endpoints were mortality, revision rate and radiographic outcome. Collected and analyzed data did comply with the recommendation of the recent systematic review of Khan et al. [14]. The electronic databases of the hospitals were retrospectively searched for patient characteristics, rate of revision surgery as well as the reason for revision.

### Secondary endpoints

Secondary endpoints were epidemiological and demographic data, such as age, gender, time to index fracture, primary implants, type of surgical procedure, American Society for Anesthesiologists (ASA) and functional outcome [14].

To quantify perioperative risk, the American Society for Anesthesiologists (ASA) score was recorded. The type of surgical approach was classified either as revision arthroplasty (RA) or open reduction with internal fixation (ORIF).

The health status was determined with the EQ-5D-5L and the functional outcome by the modified Harris-Hip-Score. Level of activity after periprosthetic femur fracture was quantified utilizing the Glasgow Outcome Score (GOS) [15].

### Radiological evaluation

Pre-operative imaging (X-rays and CT scan with metal artefact reduction) was analyzed to classify the PPF according to the Vancouver classification system and intra-operative verification of stem stability was recorded at all operative notes.

At follow-up only patients with adequate radiological datasets and a minimum follow-up time of 6 months were included for further analysis. The radiographs (X-rays and if available CT scans) were systematically analyzed and the fracture was judged as healed when callus was seen bridging the site in both the anteroposterior (AP) and lateral planes [16].

Implant fixation of the femoral stem was evaluated according to Engh et al. (bone-ingrowth fixation, stable fibrous fixation, unstable fixation) [17]. The vertical subsidence of the femoral hip stem was measured as distance between the apex of greater trochanter to the shoulder of stem in immediate postoperative and final follow-up radiograph. A subsidence up to 3 mm was graded acceptable and more than 5 mm as poor [18].

The results were graded following the criteria proposed by Beals and Tower (B&T) [19]. An excellent outcome was a stable arthroplasty and healed fracture with minimal deformity and no shortening. A stable subsidence of the prosthesis or moderate deformity/shortening was graded good. A poor result was defined as loose prosthesis, nonunion or severe shortening [19].

### Statistics

Data are presented as median (interquartile range). RStudio (RStudio Team (2020). RStudio: Integrated Development Environment for R. RStudio, PBC, Boston, MA URL http://www.rstudio.com/) was used for data processing.

Shapiro–Wilk test was performed to test for normality. The nonparametric Mann–Whitney *U* test was used to assess significant differences between two groups. To calculate the strength and association of two categorical variables, the odds ratio and 95% confidence interval was calculated. The Fisher’s Exact Test was used to assess significant differences between two categorical variables. *P* value < 0.05 was considered statistically significant.

## Results

### General data

In total, 129 patients met inclusion criteria and were available for data analysis. The median age of the study cohort was 79 years (10.3), including 90 female (70%) and 39 male (30%) patients (Table 1). Female patients had an average age of 80 (9.9) years and male patients of 78 (12) years (*p* = 0.153). The majority of the patients had an ASA score of ≥ 3 and sustained the PPF after primary hip replacement. According to the Vancouver Classification system, 20 patients sustained a Vancouver A, 90 patients a Vancouver B and 19 patients a Vancouver C fracture (Table 1).

**Table 1:** Overview of the patient characteristics and outcome data

Demographics	
Age, y, median, (IQR)	79 (10.3)
Gender	90 female (70%) 39 male (30%)
ASA score	I: 1 (2%) II: 37(29%) III: 78 (60%) IV: 10 (8%)
Primary implant	
Uncemented THA	74 (57%)
Cemented THA	37 (29%)
Cemented HA	18 (14%)
PPF after	
Primary hip replacement	104 (81%)
Revision arthroplasty	25 (19%)
The time from primary arthroplasty to PPF (mths, median, IQR)	96 (146)
Vancouver classification	
A	20 (15%)
B1	36 (28%)
B2	23 (18%)
B3	31 (24%)
C	19 (15%)

The type of treatment according to the Vancouver classification is presented in Table 2. The treatment of Vancouver A fractures involved mainly a conservative management. Vancouver B1 fractures were managed conservatively in 4 cases, 31 patients received ORIF and 1 patient RA. All Vancouver B2 patients were operatively treated, 3 with ORIF and 20 with revision arthroplasty. In patients with a Vancouver B3 fracture revision arthroplasty was performed in 27 patients and ORIF in 4 cases. All patients with a Vancouver type C fracture were treated with ORIF.

**Table 2:** Patient and outcome data of patients following ORIF in comparison to revision arthroplasty

	ORIF (*n* = 59)	Revision arthroplasty (*n* = 49)	Significance/*p* value
Age y, median, (IQR)	80 (10.5)	79 (10)	0.37
Gender	40 female (67%) 19 male (33%)	34 female (69%) 15 male (31%)	1.0
ASA (median, IQR)	3 (1)	3 (1)	0.89
Vancouver classification			
A	2 (3%)	1 (2%)	
B1	31 (53%)	1 (2%)	
B2	3 (5%)	20 (41%)	
B3	4 (7%)	27 (55%)	
C	19 (32%)	0 (0%)	
Reoperation			
	5 (8%)	14 (30%)*	< 0.05
Outcome	*N* = 14	*N* = 11	
EQ5-health VAS-score (median, IQR)	50 (45)	50 (35)	0.68
MHHS (median, IQR)	49(30)	55(28)	0.66

^*^(OR 3.4, 95% CI 1.2−10.2)

### Primary endpoints

#### Mortality

In total, 11 out of 79 patients (14%) with information regarding the final outcome died within the first 2 years after PPF. Six patients had a Vancouver A fracture, four patients a Vancouver type B fracture and one patient a Vancouver C fracture. Only one patient died during the hospital stay due to intra-operative cardiac arrest. For the other ten patients, there were no specific causes of death reported.

#### Revision rate

Overall, 107 patients were managed operatively and 20 patients (18.7%) needed reoperation. 60 patients received ORIF and 5 patients (8%) following ORIF had revision surgery due to postoperative infection (*n* = 2), stem loosening (*n* = 2) or additional/subsequent fracture (*n* = 1). In the revision arthroplasty cohort (*n* = 47), 14 patients (29.8%) had revision surgery due to postoperative infection (*n* = 8), postoperative soft tissue complications (*n* = 3), stem loosening (*n* = 2) and dislocation (*n* = 1) (OR 3.4, 95% CI 1.2–10.2) (Table 2).

#### Radiographic outcome

30 patients were available for further radiographic analysis. 17 patients received ORIF following a Vancouver B1 (*n* = 9), B2 (*n* = 1) and C (*n* = 7) fracture. 13 Patients needed revision of the stem due to a Vancouver B3 (*n* = 8), B2 (*n* = 4) and A (*n* = 1) fracture. The subsidence in final radiographs of patients following ORIF was 2 mm (2 mm) and revision arthroplasty 4 mm (5 mm). Regarding the B&T criteria, in the ORIF group, 14 patients showed an excellent outcome, 2 a poor and 1 patient a good outcome. In the revision group, eight patients revealed an excellent outcome, three a poor and two a good outcome (*p* = 0.63). According to Engh et al. implant fixation of the femoral stem in the ORIF group was graded as bone-ingrowth fixation in 14 patients, 2 times as unstable fixation and once as stable fibrous fixation. The revision group 9 patients showed a bone-ingrowth fixation, three patients an unstable fixation and one patient a stable fibrous fixation. The median follow-up time was 16 (16.5) months.

### Secondary endpoints

#### Health status and functional outcome

Self-reported health status and functional outcome was available for 25 patients. Following ORIF (*n* = 14) patients reported a median EQ5-health VAS-score of 50 (45) and after revision arthroplasty (*n* = 11) of 50 (35) (*p* = 0.68). The mHHS score was 49 (30) in the ORIF group and 55 (28) in the revision arthroplasty group (*p* = 0.66). Patients treated with ORIF showed a Glasgow Outcome Score of 5 (1) and following revision arthroplasty of 4 (1). The median follow-up time was 18 (21) months.

## Discussion

In this cohort study, 129 patients with periprosthetic femur fractures of the hip were evaluated showing that the revision rate is higher following revision arthroplasty in comparison with ORIF. Especially with respect to the complex patient population and other comparable studies, this study reports about the outcome of one of the largest numbers of periprosthetic femur fractures of the hip.

The mean age of the study cohort was 79 years and 70% were women. This is a typical age and gender distribution and also present in other comparable studies [2, 20–22]. The female preponderance might be explained by the higher lifespan and higher prevalence for osteoporosis [23, 24], contributing to a greater risk for a periprosthetic fracture of the hip [10]. More than three quarters of patients had an ASA score ≥ 3, representing a population with severe primary disease and limited overall health. Finlayson et al. analyzed the outcome following proximal periprosthetic femur fractures and reported in the majority of the patients an ASA score ≥ III [22]. Moreta et al. and Füchtmeier et al. also reported a similar risk profile for patients with PPF [24, 25]. This is an important factor that has to be considered to guide treatment since these studies showed an increased 30-day as well as 1-year mortality with increasing age and higher ASA score in patients with PPF [22, 24, 25]. As comorbidities seem to be an important variable regarding the choice for the best treatment in PPF, Patsiogiannis et al. proposed a new treatment algorithm for PPFs also taking into consideration the patient`s comorbidities [10]. This is becoming more relevant in the treatment of patients with PPF and a careful assessment of the type of fracture, functional demand and risk profile is essential to achieve the best possible outcome.

Considering the Vancouver classification system, the majority of the patients in the presented study had a Vancouver B fracture and the time from primary arthroplasty to PPF was 8 years. These results are comparable to other current studies of PPF around the hip [3, 5, 24, 26]. More than 50% of patients had a cementless femoral component, which potentially contributes a PPF independent of age and gender [27, 28]. Vancouver B fractures pose the most challenging periprosthetic femoral fracture, since the correct assessment between a stable (B1) or unstable (B2) femoral shaft can be challenging [10]. The wrong assessment of shaft stability and following inadequate surgical management can lead to reoperation due to stem loosening [14].

The 1-year mortality after periprosthetic femoral fracture is similar to that after hip fracture, ranging from 7 to 22% [3, 4, 6, 24]. In the presented study, the mortality rate of 14% is comparable, taking into consideration that the follow-up period included 2 years.

Overall, 19% of all investigated patients needed reoperation with patients following revision arthroplasty presenting a higher risk for revision surgery. The reason for revision was mostly due to a surgical site infection or soft tissue complications. Other studies reported comparable reoperation rates from 12 to19% with respect to infections or soft tissue complications [5, 14, 29]. Moreta et al. stated that fractures and infections were the most common cause for reoperation [5]. This is in line with the findings of Drew et al., who were investigating surgically treated patients with PPF [29]. The review of Khan et al. analyzed Vancouver B2 and B3 periprosthetic femur fractures and concluded, that the treatment of B2 and B3 fractures without revision of stem were associated with a higher rate of reoperation [14]. In the investigated study population, the majority of patients with a Vancouver B2 and B3 fracture were treated with revision arthroplasty. This could be one explanation for the low number of mechanical problems (i.e. stem loosening) causing revision surgery. The careful pre- and intra-operative assessment of the stem stability is crucial for guiding the right treatment and studies suggest an individual treatment especially for Vancouver B2 fractures depending on patients’ comorbidities and mobility [10, 30, 31]. Furthermore, it needs to be taken into consideration, that the patient population suffers from severe diseases with limited functional demand and a stem loosening mainly becomes manifest after a longer follow-up period. The advantage of ORIF in comparison to RA is that it poses a less invasive procedure for selected patients (Vancouver B2 fractures) [21, 32, 33].

The radiographic evaluation showed in 88% of the patients treated with ORIF and in 77% after RA, a good-to-excellent outcome according to the B&T criteria. Overall, the careful assessment and performed treatment led to a satisfactory and comparable radiographic outcome [5]. These good results in the ORIF cohort can be attributed to an exact preoperative diagnosis and differentiation between a stable and unstable femoral shaft.

Patients rated their health status and functional outcome as poor. These findings go along with other comparable studies, reporting a poor health-related quality of life, impaired walking ability and permanent pain following PPF [5, 34, 35]. The overall mHHS presents a poor functional outcome as reported by several other studies with a mean Harris-Hip Scores ranging from 40 to 67.8 [5, 32–34]. However, considering the overall poor health-related quality of life, the low mHHS score is not surprising, since physical examination is not part of the modified version.

Limitations of the study are its retrospective design with its associated bias. Only patients who were capable to give an informed consent were included, thus long-term outcomes were not available for all treated patients. As previously described, the completion of large prospective series of surgical patients of this age group is problematic [13].

Long-term clinical and radiological results were not part of our study and radiographs were taken for clinical follow-up and not for research purposes in a strict standardized manner.

The surgical procedures evaluated in this study were performed between a period of 10 years in both centers, which share the same principles of fixation and rationale of indications.

## Conclusions

Patients with a PPF of the hip are of higher age and have an increased operative risk. In case of stable prosthesis, open reduction and internal fixation results in a good radiological outcome with a low reoperation rate. Following RA, there is a higher risk for revision surgery. Although results reveal good-to-excellent clinical and radiological outcomes patients rate their own health status and the functional outcome following RA and ORIF as poor.

## Data Availability

The datasets generated during and analyzed during the current study are not publicly available due to them containing information that could compromise research participant privacy/consent, but are available from the corresponding author on reasonable request.
